# Host Factor ANP32A in Avian Influenza Virus Replication and Cross-Species Adaptation: A Narrative Review

**DOI:** 10.3390/ani16213412

**Published:** 2026-10-29

**Authors:** Zeyu Li, Shuang Zhao, Zhi Cao, Jiuzhou Song, Hongyan Sun, Qisheng Zuo, Wei Han, Guohong Chen, Bichun Li, Kai Jin

**Affiliations:** 1 Joint International Research Laboratory of Agriculture and Agri-Product Safety of Ministry of Education of China, Yangzhou University, Yangzhou 225009, China; dx120250209@stu.yzu.edu.cn (Z.L.); mz120251652@stu.yzu.edu.cn (S.Z.); dx120240182@stu.yzu.edu.cn (Z.C.); sunhy@yzu.edu.cn (H.S.); 006664@yzu.edu.cn (Q.Z.); ghchen2019@yzu.edu.cn (G.C.); yubcli@yzu.edu.cn (B.L.); 2 Key Laboratory of Animal Breeding Reproduction and Molecular Design for Jiangsu Province, College of Animal Science and Technology, Yangzhou University, Yangzhou 225009, China; 3 Institutes of Agricultural Science and Technology Development, Yangzhou University, Yangzhou 225009, China; 4 Department of Animal & Avian Sciences, University of Maryland, College Park, MD 20742, USA; songj88@umd.edu; 5 College of Bioscience and Biotechnology, Yangzhou University, Yangzhou 225009, China; 6 Poultry Institute, Jiangsu Institute of Poultry Sciences, Chinese Academy of Agricultural Sciences, Yangzhou 225003, China; hanwei830@163.com; 7 College of Biotechnology, Jiangsu University of Science and Technology, Zhenjiang 212100, China

**Keywords:** ANP32A, host factor, influenza virus, polymerase activity, host adaptation, protein–protein interaction, gene-editing

## Abstract

Highly pathogenic avian influenza virus undergoes continuous evolution with strengthened cross-species transmission capacity, posing a severe threat to global public health security. Host acidic nuclear phosphoprotein 32A (ANP32A) acts as a critical host cofactor for activating influenza viral RNA-dependent RNA polymerase, and plays a central role in viral replication and host adaptation. This review systematically elaborates the molecular features of ANP32A, as well as the mechanism by which its leucine-rich repeat (LRR) domain specifically binds to the polymerase basic protein 2 (PB2) subunit and its low-complexity acidic region (LCAR) interacts with polymerase acidic protein (PA) to facilitate the assembly and functional activation of viral polymerase complexes. It also summarizes the influences of species-specific sequence variations and splicing isoforms of ANP32A on the host restriction barrier of cross-species transmission. Further analyses reveal that ANP32A indirectly modulates the transcriptional levels of genes related to interferons, NF-κB and NOD-like receptor pyrin domain-containing 3 (NLRP3) inflammasomes via chromatin epigenetic modification and transcriptional resource competition, remodeling the cellular antiviral microenvironment as a nuclear transcriptional regulator. ANP32A and acidic nuclear phosphoprotein 32B (ANP32B) exert complementary functions to support viral polymerase activity, while acidic nuclear phosphoprotein 32E (ANP32E) only provides weak auxiliary effects on a small subset of mutant influenza strains with its primary physiological function centered on chromatin regulation; the three proteins jointly participate in the regulatory network of viral replication. Drawing on recent advances in cryo-electron microscopy structural biology and CRISPR/Cas9 gene-editing technologies, which are discussed narratively in this review, this review highlights the pivotal hub role of ANP32A in viral host adaptation and cross-species spillover.

## Simple Summary

Avian influenza viruses pose serious threats to poultry production and public health. After infecting host cells, these viruses rely on host-produced proteins to complete their replication process. The host protein ANP32A helps the influenza virus to multiply inside animal cells, and differences in this protein among different animal species can block virus transmission across species. This review describes how this host protein supports virus growth, and explains why differences in protein structure between birds and mammals affect cross-species infection. We also discuss how related family proteins work together during virus infection and their indirect influences on host immune responses. Understanding these mechanisms can provide new ideas for preventing influenza virus spillover between different animal species and developing antiviral strategies.

## 1. Introduction

In recent years, the continuous evolution of highly pathogenic avian influenza virus and the enhancement of its cross-species transmission capacity have posed persistent threats to the global poultry industry and public health security. Viruses of the H5 and H7 subtypes frequently trigger regional and transboundary outbreaks, leading to mass culling of poultry and substantial economic losses. Moreover, several viral strains have crossed interspecies barriers to infect mammals and even humans, further highlighting their potential public health risks and the urgency of prevention and control [1,2].

Avian influenza viruses are segmented, single-stranded negative-sense RNA viruses (family Orthomyxoviridae) whose replication strictly relies on the intranuclear environment of host cells and the synergistic support of diverse host factors. Among these host factors, acidic nuclear phosphoprotein 32A (ANP32A) is recognized as a critical cofactor required for the functional activation of viral RNA-dependent RNA polymerase, exerting a critical regulatory role during viral transcription and genome replication [3]. Recent studies have further revealed that avian-derived ANP32A can even be packaged into virions and facilitate the initiation of cross-species replication at the early stage of infection, indicating that ANP32A participates not only in viral replication but also in the early host adaptation of influenza viruses [4]. Existing evidence demonstrates that ANP32A interacts specifically with the core subunits of the viral polymerase complex, particularly polymerase basic protein 2 (PB2), to stabilize the polymerase conformation and activate its catalytic function. Species-specific structural variations in ANP32A constitute one of the major molecular barriers restricting cross-species transmission of avian influenza viruses [3]. In addition, distinct ANP32 family members exhibit markedly divergent capacities in modulating influenza polymerase activity, which further underscores the central role of ANP32 proteins in governing viral host range [5].

In recent years, advances in cryo-electron microscopy (Cryo-EM), single-molecule imaging and CRISPR/Cas9 gene-editing technology have driven substantial breakthroughs in research on the structural basis and functional mechanisms of ANP32A throughout the viral replication cycle. Structural biological studies have revealed that ANP32A acts as an electrostatic chaperone, which stabilizes the apo-form viral polymerase complex and facilitates its transition to an active conformation [6]. Meanwhile, gene-editing and loss-of-function models have further verified that depletion of ANP32A drastically impairs viral polymerase activity. Viruses can accumulate key mutations within PB2 and polymerase acidic protein (PA) subunits to adapt the viral polymerase for efficient utilization of mammalian ANP32A, thereby facilitating adaptive evolution in novel host environments [7]. Notably, this host-adaptation mechanism differs from the rare ANP32A-independent replication phenotype also described in ref. [7].

Nevertheless, despite the aforementioned progress, several critical questions remain to be thoroughly elucidated at the molecular level. It is still unclear how ANP32A precisely coordinates the conformational stability of the polymerase complex, mediates functional adaptation across divergent host backgrounds, and exerts systematic effects during cross-species viral transmission. Accordingly, this review systematically summarizes the structural and functional properties of ANP32A, its molecular interaction mechanisms with viral polymerases, and species-specific regulatory networks, focusing on its pivotal roles in viral host adaptation and cross-species spillover based on cutting-edge structural and functional evidence. This work aims to provide a theoretical foundation for deciphering virus–host interplay and developing ANP32A-targeted antiviral therapeutics.

## 2. Literature Search Strategy

This is a narrative review focusing on host factor ANP32A and its regulatory mechanisms during avian influenza virus replication and cross-species adaptation. Literature retrieval was performed mainly using the PubMed, Web of Science, and CNKI databases, with searches executed in June 2026. Keyword combinations for title-and-abstract searching included “ANP32A”, “avian influenza virus”, “influenza polymerase”, “host-factor”, “cross-species transmission”, “innate immunity”, and “chromatin modification”. The publication time range for database searching was set from 2005 to June 2026. Earlier seminal foundational papers published before 2005 were additionally included via reference-list chasing.

Original research articles, structural biology papers, relevant reviews, and high-quality communications were screened. Literature selection prioritized peer-reviewed publications with experimental evidence including cryo-electron microscopy structural data, gene-editing functional verification, and virus–host interaction assays. Papers with insufficient experimental evidence, duplicated reports, and conference abstracts without full-text were excluded. A total of 426 records were initially retrieved, and 54 papers were finally retained after screening. Retrieved publications were manually sorted and synthesized to summarize structural features, polymerase-supporting functions, immune-regulatory effects, and unresolved scientific questions related to ANP32-family proteins. No formal systematic review protocol or PROSPERO registration was performed for this narrative review.

## 3. Molecular Characteristics and Domain Functions of ANP32A

ANP32A acts as the core host factor mediating avian influenza virus replication. The specific molecular architecture and synergistic functions of its structural domains lay the foundation for its interaction with viral proteins and regulation of viral replication. Clarifying the molecular features and domain functions of *ANP32A* provides structural theoretical support for further elucidating the interaction mechanisms.

The protein encoded by the ANP32A gene has a molecular weight of approximately 32 kDa. Minor differences exist in the amino acid lengths of ANP32A across various species: avian ANP32A (XP_413932.3, retrieved June 2025) consists of 281 amino acids, porcine ANP32A (XP_003121807.3, retrieved June 2025) contains 249 amino acids, and murine ANP32A (NP_033802.2, retrieved June 2025) comprises 247 amino acids. ANP32A contains an N-terminal leucine-rich repeat (LRR) domain and an unstructured C-terminal low-complexity acidic region (LCAR) [8]. Early crystallographic work uncovered the core fold architecture of ANP32A protein, providing initial structural insights into its LRR-based protein-interaction interface [9]. A comprehensive early review has systematically summarized sequence features and evolutionary conservation of ANP32 protein family across eukaryotes [10]. The LRR domain of ANP32A plays a critical role in supporting influenza polymerase function. Molecular docking, co-immunoprecipitation and yeast two-hybrid assays have verified that the LRR domain of ANP32A interacts with the PB2 627 domain (residues 538–680 of PB2) [11]. The acidic LCAR domain exerts dual functions via electrostatic interactions and acts as a vital module regulating viral polymerase stability and subcellular localization. On one hand, this acidic region binds to the N-terminal segment of the viral PA subunit, generating synergistic binding effects together with the LRR-PB2 interaction to assemble the ANP32A-PB2-PA ternary complex and stabilize the overall conformation of viral polymerase [12]. The high-resolution crystal structure of influenza viral polymerase in complex with the viral RNA promoter was determined in earlier studies, laying structural groundwork for subsequent host-cofactor interaction analysis [13]. Prior research also demonstrated that pp32 could specifically boost complementary-sense RNA (cRNA)-dependent viral genome replication [14]. Apart from the well-known PB2-627 site, residues such as PB2-271 also contribute to host adaptation by modulating polymerase–host–factor interplay [15].

**Figure 1:**
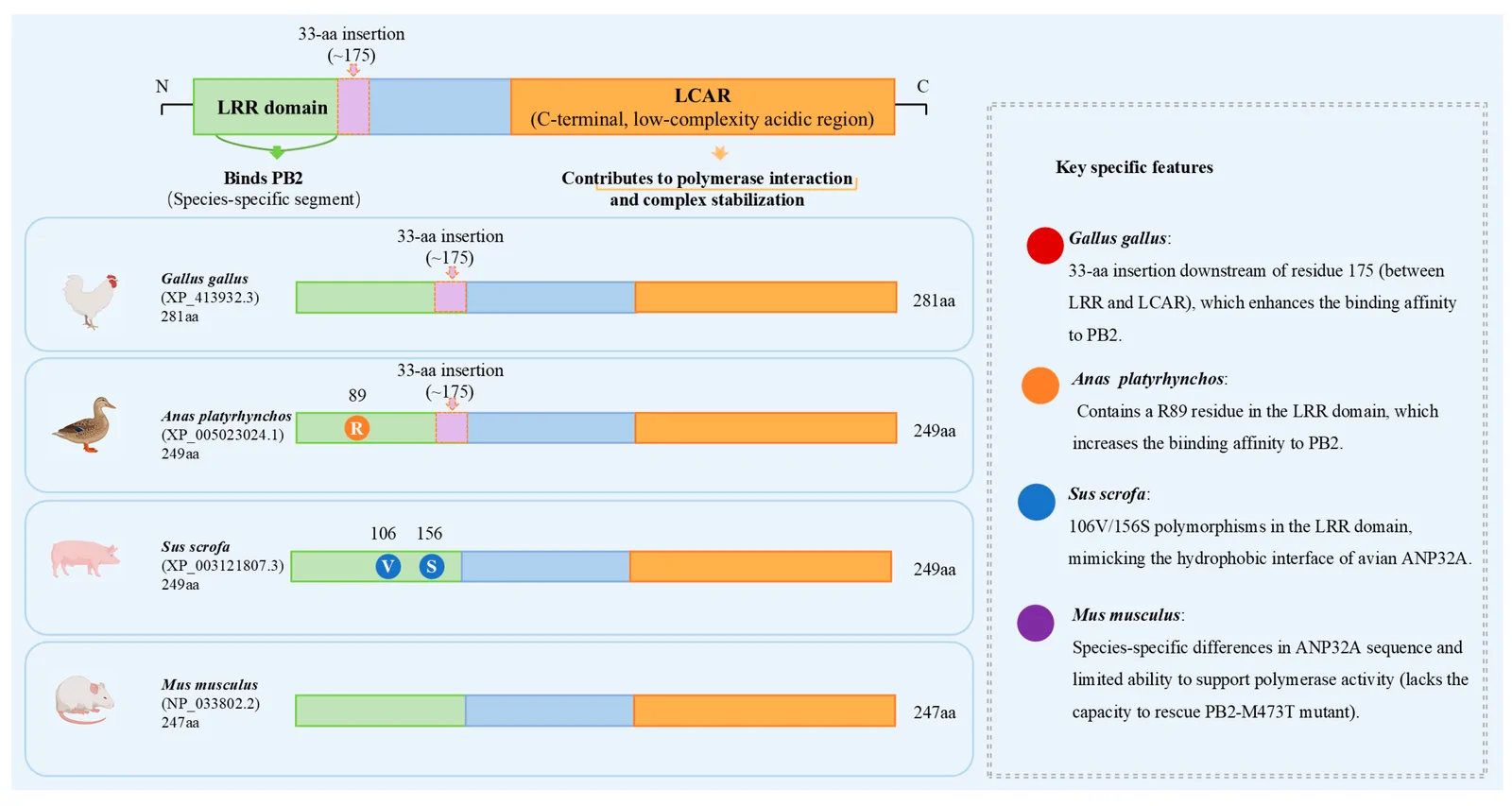
Domain composition of ANP32A from chicken (*Gallus gallus*), duck (*Anas platyrhynchos*), pig (*Sus scrofa*) and mouse (*Mus musculus*). The figure is original.

Sequence variations in ANP32A among distinct species are predominantly located within the LRR domain, which constitutes a key determinant governing the host specificity of avian influenza viruses (Table 1, Figure 1). Avian ANP32A harbours a unique 33-amino-acid insertion downstream of approximately residue 175, located between the LRR domain and LCAR; this insertion strengthens the binding stability between ANP32A and the viral PB2 subunit [16]. Porcine ANP32A carries characteristic 106V/156S polymorphisms in its LRR domain, which mimic the hydrophobic binding interface of avian ANP32A [5]. Duck ANP32A possesses a unique arginine residue at position 89; this R89 residue enhances binding affinity to the PB2 subunit of avian influenza viruses and contributes to the reservoir host characteristics of ducks [17]. Chicken ANP32A can specifically rescue the defective polymerase activity of influenza viruses bearing the PB2-M473T mutation, whereas murine ANP32A and ANP32B lack this capacity due to structural discrepancies. This finding further elucidates the molecular mechanism by which influenza viruses achieve host adaptation through specific interactions with host ANP32A [5,16]. Earlier functional assays had already indicated that human cellular environment imposed strong restriction on avian influenza polymerase activity before ANP32A was identified as the key restricting host cofactor [18]. Preliminary screening studies also pointed toward the existence of host-encoded modulators that shape avian influenza polymerase performance across different species [19].

## 4. Core Mechanisms Underlying ANP32A-Mediated Avian Influenza Virus Replication

### 4.1. Molecular Pathways for Viral Polymerase Assembly and Activation

ANP32A is an essential host factor required for the functionality of influenza viral RNA polymerase. It directly interacts with the PB2 627 domain to stabilize the polymerase complex and trigger its activation, and further facilitates polymerase dimerization to support cRNA-to-viral genomic (negative-sense) RNA (vRNA) [11,21,22]. Meanwhile, structural variations in ANP32A across different species, such as the avian-specific 33-amino-acid insertion, constitute a major molecular barrier restricting the cross-species transmission of avian influenza viruses [23,24].

Among these domains, the LRR domain is primarily responsible for specific binding to the PB2 627 domain (residues 538–680) of the PB2 subunit. This interaction facilitates the initial assembly of the polymerase complex and supports formation of a functionally competent polymerase core conformation [23,25]. Meanwhile, the LCAR of ANP32A engages in dynamic, multivalent weak interactions with the PA subunit, which modulate the spatial conformation and assembly efficiency of the polymerase complex [11,25]. Accordingly, ANP32A coordinates a network of weak interactions involving multiple subunits including PB2 and PA to drive the formation of functional viral ribonucleoprotein (vRNP) complexes. As a result, it boosts the efficiency of viral RNA transcription and replication without directly participating in catalytic reactions [23,24].

At the functional level, multiple genetic and biochemical studies have consistently demonstrated that *ANP32A* knockdown or knockout drastically impairs the transcriptional and replicative activity of influenza viral RNA polymerase, leading to greatly attenuated or nearly abolished viral propagation in diverse cellular models. Exogenous supplementation of avian ANP32A can effectively rescue polymerase function and restore viral replication capacity, further validating its essential supportive role throughout the viral life cycle [3,25]. Notably, this functional dependency exhibits prominent species specificity. Human ANP32A generally fails to fully sustain the activity of avian influenza polymerase, whereas avian ANP32A markedly boosts viral replication in mammalian cells. This discrepancy is closely linked to the adaptation matching at the PB2 627 residue [16,23]. Further mechanistic investigations reveal that loss of ANP32A predominantly shifts viral polymerase from an active transcription/replication conformation to a low-activity state, drastically reducing the synthesis of both vRNA and cRNA. This indicates that ANP32A not only acts as an on/off switch for polymerase activation but also sustains continuous RNA synthesis by stabilizing the overall polymerase complex [3,25]. Collectively, ANP32A serves as a canonical host dependency factor at the functional level, and simultaneously acts as a critical molecular checkpoint governing influenza viral cross-species adaptation and replication efficiency.

**Figure 2:**
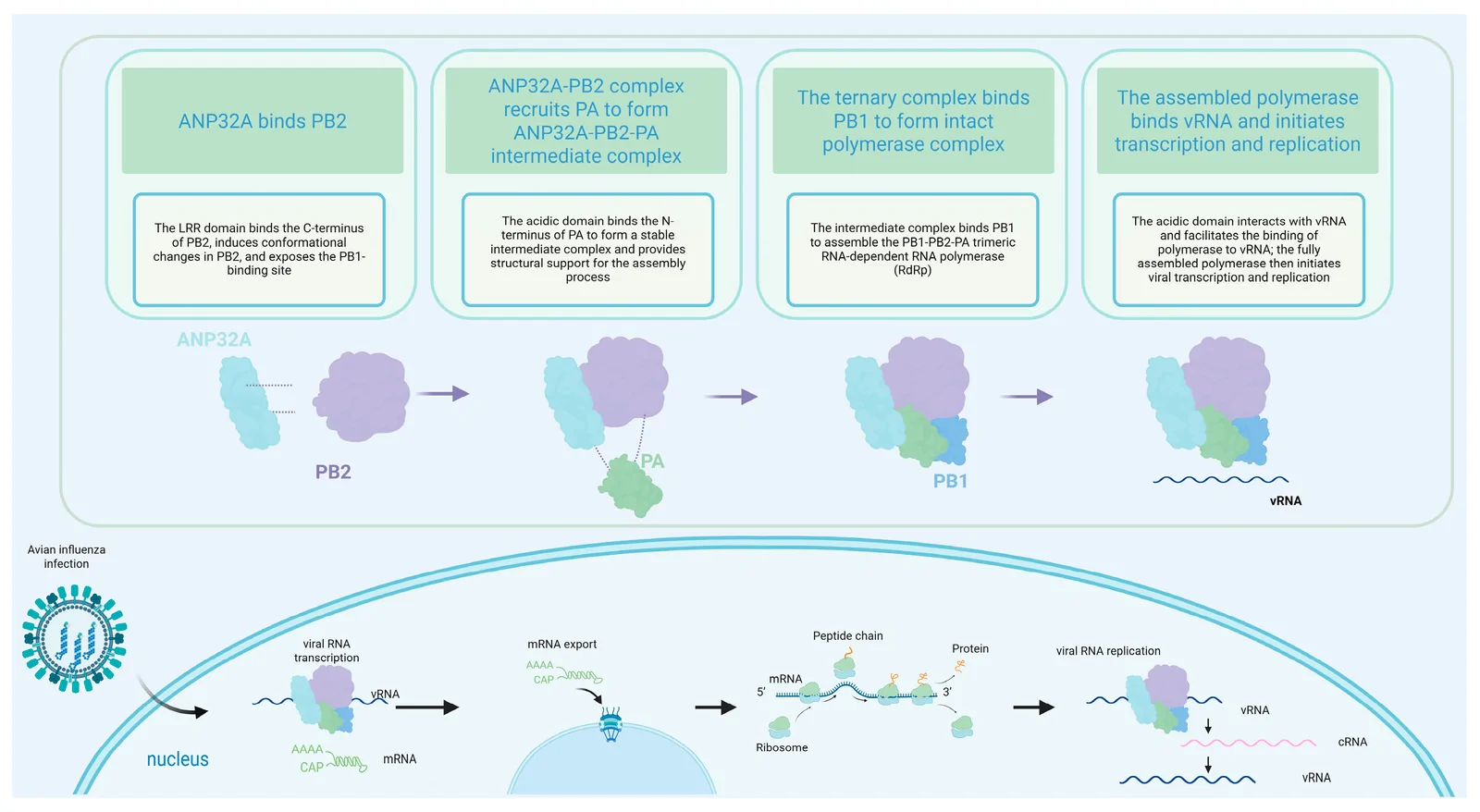
Schematic diagram illustrating ANP32A-mediated assembly of avian influenza virus polymerase and subsequent viral transcription and replication within host cells. Colour coding: light blue, host ANP32A protein; purple, PB2 subunit of influenza viral polymerase; green, PA subunit; dark blue, PB1 subunit; dark blue wavy line, viral genomic vRNA; pink wavy line, complementary cRNA; black circle icon, avian influenza virion; Arrow definition: solid purple and black arrows represent sequentially occurring molecular events that are experimentally validated; dotted grey lines denote direct protein–protein interaction interfaces between molecules; This schematic summarizes the stepwise assembly of the influenza trimeric RNA-dependent RNA polymerase (RdRp) complex assisted by host ANP32A. After full assembly, the polymerase binds vRNA to initiate viral transcription and replication in the host nucleus, generating capped mRNA for cytoplasmic protein translation and cRNA intermediates for vRNA amplification. Note: All core protein assembly steps in the upper panel have been experimentally verified. The downstream transcription, mRNA export, translation and vRNA replication cycle in the lower panel represent the well-established canonical replication pathway of avian influenza virus. The figure is original.

In summary, ANP32A facilitates the assembly and activation of the viral polymerase complex via LRR-mediated binding to PB2, LCAR-associated interactions with PA, and scaffolding of the ternary complex. Structural variations in ANP32A among different species further establish a crucial regulatory checkpoint restricting cross-species transmission. This mechanism not only unveils the molecular basis underlying the dependence of influenza viruses on host factors for replication, but also provides an important theoretical framework for understanding viral cross-species adaptation and evolution (Figure 2).

### 4.2. Regulation of Signaling Pathways Associated with Viral Replication

During influenza virus replication, ANP32A not only functions as an essential host cofactor for viral RNA polymerase, but may also indirectly modulate the assembly and functional status of vRNP complexes by integrating the host cellular protein modification network and the intranuclear transport environment. The host SUMOylation (conjugation of small ubiquitin-like modifier) pathway broadly regulates the stability and transcriptional activity of intranuclear protein complexes during viral infection. Influenza virus infection triggers dynamic alterations in host small ubiquitin-like modifier (SUMO)-associated modification levels, thereby reshaping the microenvironment supporting viral replication [26,27].

Based on current limited evidence, we propose a hypothetical model: Against this backdrop, it is hypothesized that ANP32A may indirectly participate in the dynamic regulation of vRNP-associated protein complexes by modulating polymerase complex assembly stability. ANP32A/B SUMO-modification could be hijacked by viral NS2 to promote vRNP replication competence [27]. ANP32A also exhibits functional crosstalk with NS2-mediated vRNP nuclear export, potentially regulating the nucleocytoplasmic transport of viral genomic RNA [23,25]. Although whether ANP32A directly interacts with viral proteins through SUMO-interacting motifs (SIMs) remains to be experimentally verified, available evidence supports the hypothesis that the ANP32A-related host protein network may improve vRNP assembly and function by shaping the intranuclear environment.

At the level of polymerase function, ANP32A primarily modulates RNA synthesis efficiency through species-specific interactions with the PB2 subunit. Adaptive mutations at critical PB2 residues (such as position 627) enable the polymerase to efficiently utilise mammalian ANP32A, consequently enhancing viral replication capacity in mammalian cells [23,24]. Accordingly, ANP32A may promote the stable formation of vRNP complexes and elevate viral replication efficiency via a dual mechanism comprising direct regulation of polymerase function and indirect modulation of the intranuclear environment.

## 5. Functional Characteristics and Interaction Networks of ANP32 Family Homologous Proteins

### 5.1. Functional Complementation and Divergence of Homologous Proteins

The acidic nuclear phosphoprotein 32 (ANP32) family consists of a group of highly conserved nuclear-localized proteins, mainly including ANP32A, ANP32B and acidic nuclear phosphoprotein 32E (ANP32E). Proteins of this family exert vital functions in transcriptional regulation and chromatin-related processes, such as modulation of histone acetylation and transcriptional complex regulation, and participate in diverse cellular biological processes [28,29]. During influenza virus replication, ANP32A and ANP32B serve as complementary essential cofactors, whereas ANP32E only weakly sustains polymerase activity of a small number of mutant viral strains and primarily mediates cellular chromatin remodeling, reflecting prominent functional divergence among family members. Studies on influenza A virus (IAV) have demonstrated that viral polymerase activity is markedly reduced and viral replication is severely impaired in cells with double deletion of *ANP32A* and *ANP32B*, indicating that ANP32A and ANP32B possess critical and partially redundant functions in supporting IAV polymerase activity [30]. By contrast, influenza B virus exhibits distinct dependency patterns on ANP32 family proteins. In cell models with individual or combined deletion of *ANP32A*, *ANP32B* and *ANP32E*, ANP32A and ANP32B remain the predominant host factors supporting influenza B virus polymerase activity. ANP32E only confers weak supportive capacity and fails to sustain efficient viral replication under double or multiple gene deletion backgrounds [31]. These findings reveal that ANP32 family members display functional division and divergent supporting efficiencies across different influenza virus subtypes.

Furthermore, splice variants and structural variations in avian ANP32A may affect the efficiency of its interaction with the viral polymerase complex, resulting in divergent viral RNA synthesis capacity in different host cells. Such differences may, to a certain extent, influence viral replication fitness and host restriction profiles across various host cell types [32].

### 5.2. Novel Findings on Interaction Networks of Homologous Proteins

In recent years, proteomic and virus–host interaction studies have revealed that ANP32 family proteins may participate in the regulation of influenza virus replication by forming multi-layered protein interaction networks. Within the family, ANP32A and ANP32B exhibit partial functional overlap in the cell nucleus and exert synergistic supportive effects on influenza viral polymerase activity in certain experimental systems. Nevertheless, conflicting opinions remain regarding whether they function in the form of stable homodimers or heterodimers: one set of evidence supports that ANP32 proteins form stable dimeric complexes to assist viral polymerase function [21], while other studies demonstrate that monomeric ANP32 proteins are sufficient to activate the viral polymerase complex [8,31]. Such discrepancies may stem from variable experimental set-ups, protein abundance and detection techniques among different studies. At present, it remains unclear whether ANP32 homodimer or heterodimer formation is an essential physiological prerequisite for supporting influenza viral polymerase activity. In contrast, owing to differences in domain architecture and sequence features, ANP32E displays weak activity in supporting influenza viral polymerase, and its functions are primarily associated with host chromatin regulation and transcription-related processes. In terms of interactions outside the ANP32 family, as factors associated with the inhibitor of histone acetyltransferases (INHAT) complex, ANP32A/B can modulate E1A-binding p300/CREB-binding protein (p300/CBP)-mediated histone acetylation, thereby altering host chromatin accessibility and the transcriptional regulatory environment. Such changes in transcriptional status may indirectly affect the allocation of host cellular transcriptional resources during viral replication and, to some extent, govern the efficiency of viral RNA synthesis [28].

**Figure 3:**
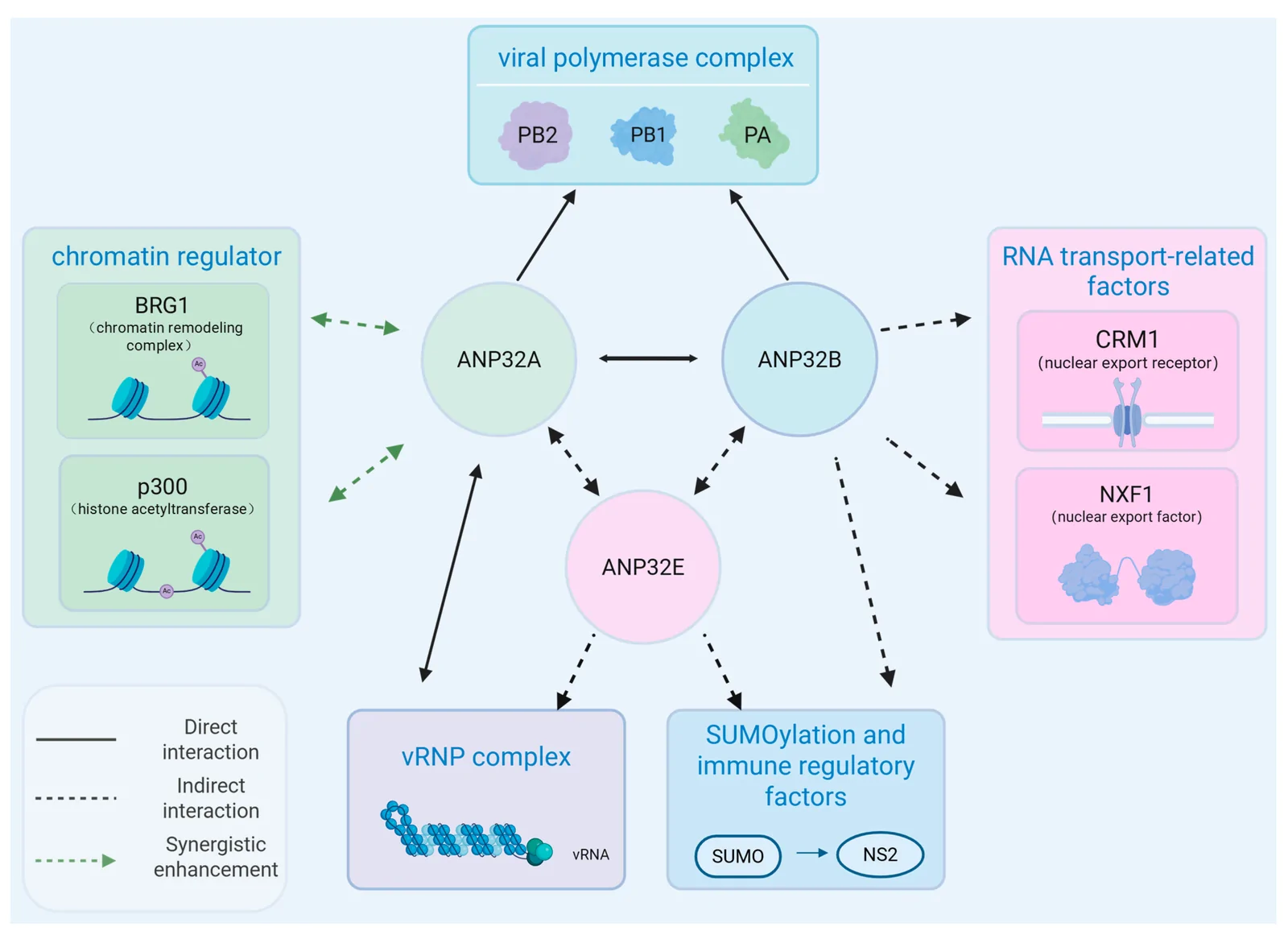
Interaction network of ANP32 family homologous proteins. Solid black lines show experimentally validated direct interactions; dashed black lines represent putative indirect interactions, and green dotted lines indicate synergistic enhancement effects. The figure is original.

Furthermore, avian ANP32A possesses prominent species-specific structural features. The approximately 33-amino-acid insertion located between its LRR and LCAR domains is recognized as one of the key structural determinants enabling its capacity to support influenza viral polymerase activity. This insertion improves its compatibility with the PB2 627 domain, facilitating stable assembly and functional activation of the viral polymerase complex [16,21]. Meanwhile, avian *ANP32A* generates multiple splice variants. These variants may compromise the integrity or spatial conformation of critical structural regions, reducing their ability to support viral polymerase and further contributing to divergent viral replication capacity in distinct host cells. Collectively, these structural variations and alternative splicing events constitute the ANP32A-mediated host restriction barrier, representing one of the crucial molecular factors governing cross-species transmission and host adaptation of avian influenza viruses [32]. Alternative splicing isoforms of avian *ANP32A* exhibit distinct potency to support influenza polymerase activity, further expanding the functional diversity of host factors within avian cells [33]. Cross-species comparison also revealed substantial functional divergence of *ANP32* orthologs in modulating viral polymerase function [34]. Under selective pressure, influenza virus could acquire adaptive mutations to gain capacity for utilizing ANP32E as alternative co-factor [22] (Figure 3).

## 6. Putative Roles of ANP32A in Modulating Host Innate Immunity

### 6.1. Inhibitory Effects on Innate Immune Pathways

As an important host factor utilized by avian influenza viruses to modulate host innate immune responses, ANP32A may participate in the regulation of interferon (IFN) and nuclear factor-κB (NF-κB)-associated signaling pathways via a multi-layered molecular interaction network. This reshapes the cellular antiviral state and generates a favorable microenvironment for viral replication. Within the type I interferon (IFN-I) signaling cascade, ANP32A indirectly governs host antiviral responses through multiple mechanisms. On one hand, as an essential host cofactor of the influenza viral polymerase complex, ANP32A predominantly localizes to the nucleus and engages in viral RNA transcription and replication, thereby altering the allocation of host cellular transcriptional resources [21]. On the other hand, ANP32 family proteins are implicated in chromatin remodeling and transcription-regulatory complexes. Changes in their functional status may modulate the expression of interferon-stimulated genes (ISGs), indirectly tuning antiviral responses at the transcriptional level [35]. For the canonical type I interferon signaling axis comprising retinoic acid-inducible gene-I (RIG-I), mitochondrial antiviral signaling protein (MAVS), tumor necrosis factor receptor-associated factor 3 (TRAF3), and interferon regulatory factor 3 (IRF3). To our knowledge, existing biochemical and structural studies have not identified stable, direct physical interactions between ANP32A and MAVS or TRAF3, indicating that ANP32A does not directly block the assembly of this signaling complex. The suppressive effect of ANP32A on IFN responses is mediated indirectly. First, abundant nuclear translocation of ANP32A facilitates viral polymerase assembly, sequestering host transcriptional machinery and limiting transcription of interferon-related genes. Second, as a component of the INHAT complex, ANP32A represses the histone acetyltransferase activity of p300 and reduces chromatin accessibility. This downregulates the transcription of ISGs and IFN-α/β at the chromatin level, ultimately attenuating the cellular antiviral state. As components of INHAT complex, pp32 could also serve as a transcriptional-machinery recruiter for ISG gene activation under certain contexts [36]. Ubiquitination modifications are critical for fine-tuning RIG-I-MAVS-dependent innate immune signaling cascades [37]. Dynamic abundance profiles of ANP32 family members further influence host antiviral status upon virus infection [38]. Host RNA regulatory machineries targeting AU-rich RNA elements can also reshape cellular responses during virus infection [39].

Beyond canonical signal transduction, ANP32A may indirectly modulate the expression of interferon (IFN)-related genes by participating in epigenetic and transcriptional regulation. Studies have demonstrated that ANP32A, as a vital component of the INHAT (inhibitor of histone acetyltransferases) complex, interacts with histone acetyltransferases including p300/CBP and suppresses their HAT activity. It further governs chromatin acetylation levels and global chromatin conformational accessibility via the histone masking mechanism [28]. With respect to the NF-κB signaling pathway, which involves IκB kinase complex (IKK), inhibitor of κB α (IκBα), p65/p50 heterodimer, TANK-binding kinase 1 (TBK1), pro-inflammatory cytokines interleukin-6 (IL-6) and tumor necrosis factor-α (TNF-α), previous reports indicate that ANP32A is involved in the assembly of transcriptional regulatory complexes and interacts with multiple transcription factors and chromatin-associated proteins, thereby altering the expression of NF-κB-dependent genes. Nevertheless, consistent evidence remains lacking to confirm whether ANP32A directly regulates the nuclear translocation of the NF-κB p65 subunit or upstream signaling events. Accordingly, ANP32A is more likely to indirectly control the expression of downstream inflammatory genes of the NF-κB pathway by shaping the transcriptional environment and chromatin status, rather than directly targeting canonical signaling nodes [35]. Overall, ANP32A functions more plausibly as a modulator operating at the transcriptional and chromatin levels, instead of acting as a core regulatory protein within classical signaling cascades in innate immune regulation. Notably, current biochemical and structural evidence does not support direct physical engagement of ANP32A with key signal-transducing molecules of innate immune cascades. Its immunomodulatory outputs are predominantly achieved through indirect epigenetic and transcriptional rewiring, rather than via direct binding to signal-transduction components to alter complex assembly.

Furthermore, ANP32A derived from distinct species exhibits marked structural divergence. Amino acid sequence variations within its C-terminal acidic region and LRR domain can substantially alter its binding capacity to the influenza viral polymerase complex, leading to species-specific differences in the efficiency of supporting viral polymerase activity [8,23].

### 6.2. Regulation of Inflammasome Activation

The inflammasome is an important multiprotein complex that initiates innate immune responses upon host cell recognition of pathogen infection. Its assembly drives caspase-1 activation via autoproteolysis, which further promotes the maturation and release of pro-inflammatory cytokines such as IL-1β and IL-18, and induces pyroptosis. Among various inflammasomes, the NOD-like receptor pyrin domain-containing 3 (NLRP3) inflammasome is one of the most extensively investigated. Previous studies have verified that it can be activated during influenza A virus (IAV) infection and participates in regulating the dynamic balance between viral replication and host inflammatory responses [40,41,42]. Activation of the NLRP3 inflammasome is coordinately modulated by multiple host and viral factors. Existing research demonstrates that influenza viruses can trigger NLRP3 inflammasome activation via mechanisms including the M2 ion channel, thereby initiating caspase-1-dependent inflammatory responses [43]. Meanwhile, numerous intracellular host regulators are likely involved in tuning the activation threshold of the inflammasome.

Within this regulatory network, ANP32A may modulate inflammation-associated immune responses through multi-layered mechanisms. Current evidence favors the notion that ANP32A acts primarily via indirect regulation, rather than directly participating in the assembly of the NLRP3 inflammasome complex. Structural studies on NLRP3 inflammasome activation have revealed that its assembly mainly relies on NACHT domain-mediated oligomerization and recruitment of the apoptosis-associated speck-like protein containing a CARD (ASC) adaptor protein. Most upstream regulators exert their effects indirectly through ion homeostasis, reactive oxygen species (ROS) signaling, transcriptional regulation and other pathways [44,45]. Furthermore, structural biological and biochemical evidence supporting stable direct binding between ANP32A and the NACHT domain of NLRP3 or ASC protein remains absent. Accordingly, ANP32A is likely to modulate inflammasome activation indirectly by shaping the host transcriptional regulatory network and cellular stress status.

At the transcriptional regulatory level, ANP32A acts as a crucial intranuclear regulatory protein and has been confirmed to participate in modulating histone acetylation status, thereby shaping downstream gene expression programs. Studies have demonstrated that ANP32A contributes to the regulation of chromatin status by tuning the acetylation level of histone H3, which further affects cell proliferation and transcriptional activity [28,29]. In addition, the ANP32A-mediated chromatin regulatory network may remodel the global transcriptional environment of host cells, indirectly interfering with multiple immune regulatory processes including inflammation-related signaling pathways.

At the cellular stress level, ROS have been validated as one of the essential upstream signals driving NLRP3 inflammasome activation [46]. Studies suggest that ANP32A may participate in the regulation of cellular stress homeostasis [47]. Nevertheless, the precise molecular mechanism by which ANP32A modulates inflammasome activation via the ROS pathway remains to be further elucidated; hence, this regulatory model is currently speculative.

Notably, during AIV infection, the virus itself can modulate host innate immune responses via diverse mechanisms. Studies have revealed that influenza viruses employ their encoded proteins to regulate multiple immune responses, including type I interferon and inflammasome-associated signaling pathways, thereby attenuating host antiviral defenses [48]. ANP32A-associated regulatory effects may cooperate with viral proteins to shape the magnitude of inflammatory responses and immune homeostasis.

**Figure 4:**
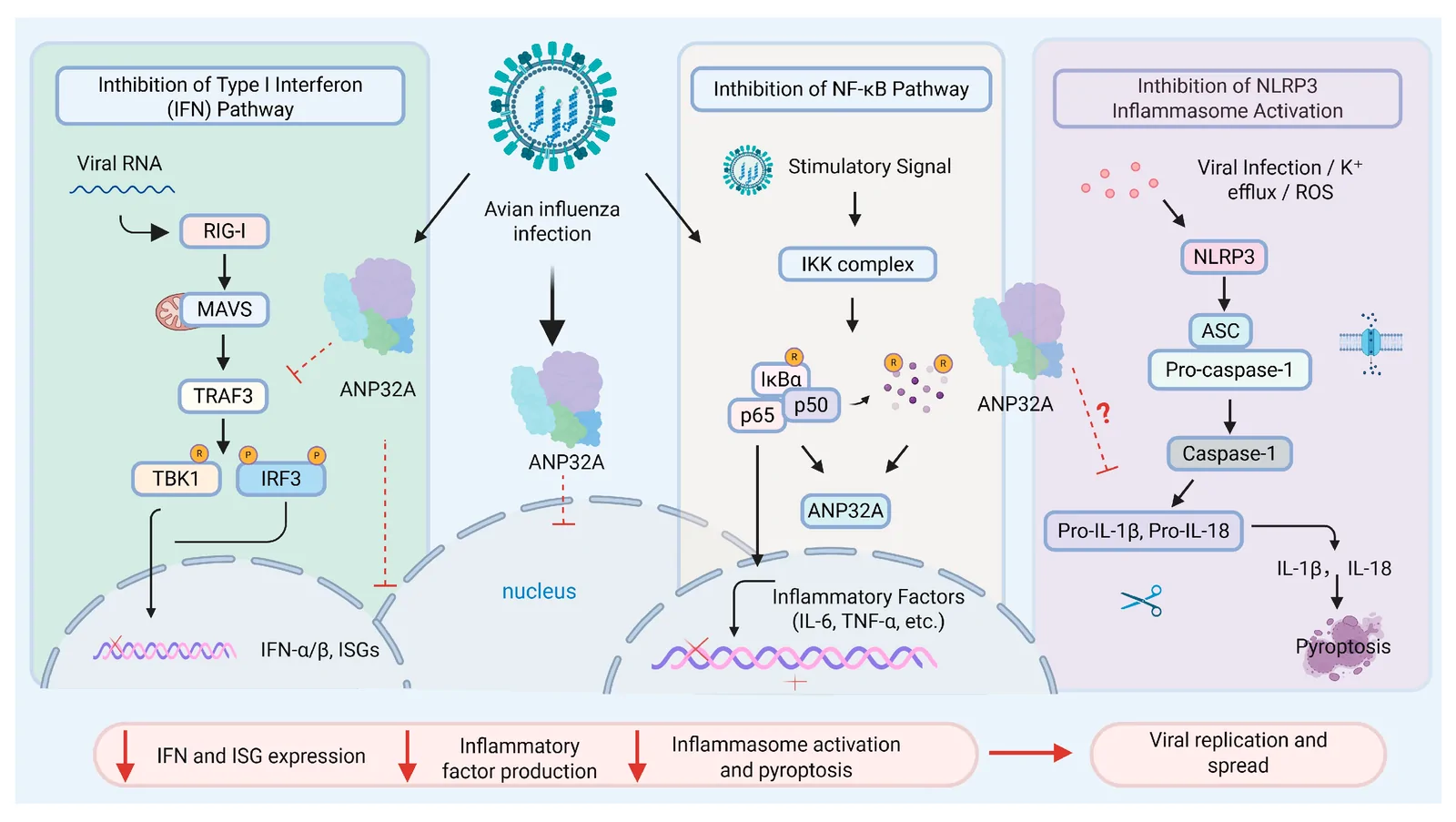
Schematic illustrating putative roles of ANP32A in modulating host innate immune signaling during avian influenza virus infection. Solid arrow: established signaling cascade; dashed inhibitory line: indirect regulatory effect; dashed inhibitory line with question mark: speculative regulatory effect (mechanism remains unclear, requires further validation). The figure is original.

Collectively, ANP32A may indirectly modulate the activation level of the NLRP3 inflammasome through multiple routes including transcriptional regulation, chromatin status modulation and cellular stress responses, thereby governing host antiviral inflammatory reactions and pyroptosis to a certain extent. Such multi-layered regulation may help maintain the dynamic balance between inflammatory responses and viral replication during infection. Nevertheless, the precise molecular mechanisms require further in-depth investigation (Figure 4).

## 7. Research Perspectives and Key Scientific Questions

### 7.1. Unresolved Core Mechanistic Questions

Although relatively systematic understanding has been established regarding the roles of ANP32A in regulating avian influenza virus (AIV) RNA polymerase function and host adaptation, several critical scientific questions at the molecular level remain unanswered. The functional divergence of ANP32 family homologous proteins across different species and the mechanisms underlying their selective dependence on polymerases of distinct AIV subtypes have not been fully clarified, and systematic dissection of the structural adaptation rules involved in cross-host transmission is still lacking. The key interaction interface between ANP32A and the AIV polymerase complex, particularly the dynamic conformational regulation of the PB2 627 domain and its adjacent regions during viral RNA transcription and replication, lacks supporting evidence from high-resolution structural and dynamic studies. Furthermore, the molecular mechanisms by which influenza viruses acquire the capacity to utilize mammalian ANP32A via mutations at key PB2 residues (such as E627K and D701N) remain incompletely elucidated. The phenomenon of ANP32A-independent viral replication represents a separate scenario and requires independent investigation. Whether such mutations are accompanied by conformational rearrangement of the polymerase complex and switching between transcriptional and replicative functions requires further verification. Finally, systematic experimental evidence is still absent to confirm whether cross-regulation or synergistic effects exist between the multi-layered regulatory networks in which nuclear ANP32A participates—including chromatin modification control and inflammation-related signaling pathways—and its canonical function during viral RNA synthesis.

### 7.2. Future Research Directions

Based on current research progress, further in-depth investigations on ANP32A can be carried out in multiple directions, including structural mechanism dissection, characterization of host adaptation rules, and development of antiviral strategies. At the structural mechanistic level, Cryo-EM, single-molecule imaging and computational structural biology approaches can be combined to resolve the dynamic structures of ANP32A in complex with the AIV polymerase complex, so as to define the conformational regulatory basis underlying viral RNA transcription and replication. In terms of host adaptation, long-term serial passaging models and high-throughput sequencing technologies can be utilized to systematically map the lineages of viral adaptive mutations driven by altered ANP32A dependence, thereby illuminating evolutionary trajectories and selective pressures during cross-species transmission. From a systems biology perspective, multi-omics datasets including transcriptomic, proteomic and interactomic data should be integrated to dissect functional differences in ANP32 family proteins across various tissues and cellular microenvironments, as well as their regulatory characteristics governing viral replication efficiency, enabling the construction of a framework for host-dependent regulatory networks. For translational applications, targeted intervention strategies can be designed based on the key functional domains of ANP32 family proteins. Combined with gene-editing technology, avian influenza-resistant animal models can be established. Meanwhile, structure-based molecular intervention approaches can be explored to provide theoretical foundations for broad-spectrum anti-influenza strategies. Novel host-adaptation sites such as PB2-M473T in H7N9 expand our understanding of how influenza viruses exploit ANP32 proteins for cross-species spillover [49]. Gene-editing targeting *ANP32* family has demonstrated promising proof-of-concept for generating avian-influenza-resistant poultry [50]. Nevertheless, several translational risks remain to be carefully considered before practical application of ANP32-targeted gene-editing strategies. Off-target modification represents a primary technical concern, which may trigger unintended disruption of host genome and perturb normal physiological functions mediated by endogenous ANP32 family proteins. In addition, ANP32 family participates in multiple essential cellular processes beyond viral replication; gene manipulation may compromise animal fitness, growth performance or immune homeostasis. Furthermore, the possibility of generating viral escape mutants under host-gene editing pressure should not be ignored. Therefore, comprehensive biosafety evaluation, phenotypic monitoring and risk-benefit assessment are required prior to real-world deployment of such gene-edited avian models. Beyond virus infection, ANP32A also participates in maintaining cellular chromatin homeostasis and oxidative-stress responses [48]. Targeted peptide-based intervention strategies against ANP32A-related pathways have been explored in other disease models, which may inspire anti-influenza therapeutic development [51]. Insights gained from inflammasome-associated human-disease research also provide reference for interpreting avian-host inflammatory responses [52].

## 8. Conclusions

Accumulating studies have demonstrated that host ANP32 family proteins, especially ANP32A, serve as central host co-factors for avian influenza virus polymerase. The species-specific 33-amino-acid insertion located between the LRR and LCAR domains of ANP32A constitutes the principal molecular determinant governing its capacity to support viral polymerase activity and shapes the molecular barriers for cross-species spillover of avian influenza viruses. Beyond assisting viral replication, ANP32A also remodels host antiviral microenvironment via chromatin epigenetic regulation and indirectly modulates interferon, nuclear factor-κB and NLRP3 inflammasome-related immune responses. ANP32A, ANP32B and ANP32E form a complicated regulatory network, exhibiting partial functional compensation and divergence upon influenza virus infection. Nevertheless, multiple critical molecular puzzles remain unaddressed, including dynamic conformational regulation during polymerase-ANP32A interaction, crosstalk between its polymerase-supporting function and chromatin-related cellular roles. Further structural biology, multi-omics and gene-editing work will deepen our understanding of virus–host interplay and inspire new anti-influenza intervention strategies.

Limitations of this review: This review is based on a non-systematic literature search without formal quality appraisal of included studies. Most functional evidence comes from cell-based minireplicon and polymerase assays, while in vivo infection data remain relatively scarce.

## Figures and Tables

**Table 1:** Species-specific sequence variations in the ANP32A LRR domain and their impacts on interactions with the AIV PB2 subunit and viral host specificity.

Species Type	Core Sequence Variations in the LRR Domain	Interaction Effects with Avian Influenza Virus PB2 Subunit	Regulatory Roles in Viral Host Specificity	References
Avian (Chicken) XP_413932.3	Possesses a unique 33-amino-acid insertion downstream of approximately residue 175 between the LRR domain and LCAR (residue numbering refers to XP_413932.3).	This insertion provides a hydrophobic and SUMO-interaction-like surface for PB2, conferring high binding affinity and supporting the polymerase activity of PB2-627E variants.	As an adaptive host factor for natural avian influenza reservoirs, it guarantees efficient viral replication in avian hosts.	[16]
Avian (Duck) XP_005023024.1	Contains a 33-amino-acid insertion downstream of residue 175 between the LRR domain and LCAR, and bears arginine (R) at position 89 (residue numbering refers to XP_005023024.1).	The R89 residue enhances binding affinity to the PB2 subunit compared with chicken-derived ANP32A.	It contributes to host adaptability, making ducks natural reservoir hosts for avian influenza viruses.	[17]
Porcine XP_003121807.3	No 33-amino-acid insertion; carries specific 106V/156S polymorphisms (residue numbering refers to XP_003121807.3).	The 106V/156S motif mimics the hydrophobic binding interface of avian ANP32A.	As an intermediate host adaptation factor, it permits the accumulation of adaptive viral mutations such as PB2-E627K.	[5]
Murine NP_033802.2	No 33-amino-acid insertion; key residues within the LRR 129–130 region mediate restriction phenotype (residue numbering refers to NP_033802.2).	It fails to rescue the defective activity of PB2-M473T mutant polymerase; binding to the PB2 subunit cannot effectively initiate viral RNA synthesis.	It imposes strong restriction on avian influenza virus replication; PB2-D701N is a naturally occurring mammalian-adaptive mutation that partially overcomes this restriction.	[20]
Human NP_006296.1	Lacks the 33-amino-acid insertion (residue numbering refers to NP_006296.1).	Restricts the polymerase activity of avian-origin PB2.	Limits cross-species spillover of most avian influenza strains into human hosts.	[16]

## Data Availability

No new data were created or analyzed in this study. Data sharing is not applicable to this article.

## Abbreviations

The following abbreviations are used in this manuscript:

ANP32A	Acidic nuclear phosphoprotein 32A
ANP32B	Acidic nuclear phosphoprotein 32B
ANP32E	Acidic nuclear phosphoprotein 32E
PB2	Polymerase basic protein 2
PA	Polymerase acidic protein
LRR	Leucine-rich repeat
LCAR	Low-complexity acidic region
NLRP3	NOD-like receptor pyrin domain-containing 3
vRNA	Viral genomic (negative-sense) RNA
cRNA	Complementary-sense RNA
vRNP	Viral ribonucleoprotein
SUMOylation	Small ubiquitin-like modifier modification
SUMO	Small ubiquitin-like modifier
NS2	Non-structural protein 2
IAV	Influenza A virus
INHAT	Inhibitor of histone acetyltransferases
p300/CBP	E1A-binding p300/CREB-binding protein
ISGs	Interferon-stimulated genes
MAVS	Mitochondrial antiviral signaling protein
TRAF3	Tumor necrosis factor receptor-associated factor 3
IRF3	Interferon regulatory factor 3
RIG-I	Retinoic acid-inducible gene-I
IFN	Interferon
IFN-I	Type I interferon
NF-κB	Nuclear factor-kappa B
Cryo-EM	Cryogenic electron microscopy
HAT	Histone acetyltransferase
SIMs	SUMO-interacting motifs
IL-1β	Interleukin-1β
IL-18	Interleukin-18
ASC	Apoptosis-associated speck-like protein containing a CARD
ROS	Reactive oxygen species
AIV	Avian influenza virus
NXF1	Nuclear export factor 1
BRG1	Brahma-related gene 1
